# Sociodemographic predictors of beliefs about getting HIV infection by witchcraft or supernatural means: a population-based study of 15335 Senegalese women

**DOI:** 10.4314/ahs.v24i1.6

**Published:** 2024-03

**Authors:** Amr Ehab El-Qushayri, Amira Yasmine Benmelouka

**Affiliations:** 1 Faculty of Medicine, Minia University, Minia 61519, Egypt; 2 Faculty of Medicine, University of Algiers, Algeria

**Keywords:** HIV, infection, witchcraft, misconception, myths

## Abstract

**Aim:**

To provide more insights about beliefs of witchcraft and supernatural means as causes of human immunodeficiency virus (HIV) among women in Senegal.

**Method:**

We included eligible women from the demographic and health survey conducted in Senegal during the year 2017.

**Results:**

We included 15335 women, of those 620 (4%) thought that they can get HIV through witchcraft or supernatural means. After the adjustment of all available covariates, old age, receiving primary or secondary education, higher wealth index, more frequency of listening to radio, watching television for less than once a week and reading newspaper or magazine for at least once a week were significantly associated with a reduction in the witchcraft and supernatural means beliefs (p < 0.05). Moreover, rural residence was associated with an increase in the wrong HIV beliefs (p < 0.05).

**Conclusion:**

We demonstrated many predictors of the wrong beliefs about getting HIV infection by witchcraft or supernatural means in the Senegalese women. Policymakers should initiate health educational programs in parallel with increasing the socioeconomic status to limit the HIV transmission. In addition, continuous monitoring of the HIV knowledge in the endemic countries is crucial to decrease HIV burden.

## Introduction

According to the World Health Organization (WHO) in 2020, the prevalence of the human immunodeficiency virus (HIV) was nearly 38 million patients including 1.5 million newly infected patients 1. The prevalence of HIV patients was much higher in African countries rather than other continents, with the main contribution of sub-Saharan countries in such high prevalence 2. Due to the high prevalence and the multiple ways of infection transmission, a breakthrough in HIV treatment occurred in the mid of the 20^th^ century with the discovery of antiretro-viral therapy (ART) and a substantial decline in mortality rates was noticed 3.

Knowledge about HIV constitutes one of the major barriers for limiting its transmission. However, in many sub-Saharan countries there is a gap of knowledge about HIV resulting in HIV stigma, witchcraft beliefs and transmission myths 4, 5. Despite the remarkable technological improvement, the thought of getting HIV infection through witchcraft or supernatural means beliefs still exists 6. A South African study indicated that 11% of the recruited participants believed about getting HIV infection through spirits/supernatural means 5. Moreover, 6% of the Kenyan individuals caring for one or more HIV family member, thought that witchcraft is one of the causes of HIV 6. In a population-based study in Ghana, women reported a higher prevalence regarding the misconception of getting HIV through witchcrafts rather than men, 31% and 29%, respectively 4. Herein, we aimed to study the prevalence and the sociodemographic predictors for beliefs of getting HIV through witchcrafts and supernatural means in Senegal women.

## Method

### Data source

We extracted the needed data from the Demographic Health and Survey (DHS) of Senegal that was conducted in 2017. The survey contained the data regarding attitudes and beliefs about HIV transmission. Herein, we included all eligible women who reported information about beliefs of witchcraft or supernatural means of getting HIV. The question was included in the database as the following “Can get HIV by witchcraft or supernatural means?” All women who reported a yes answer were included as a case group and were encoded in the DHS database as code 1, while all women who reported a no answer were included as controls and were encoded as code 0. We excluded all women with missing data and women with a “do not know” answer to the question of interest.

### Ethical consideration

A request for data usage was approved from the DHS board on 13^th^ March 2022. Before participation in the survey, informed consent is read to the participants regarding the purpose of the survey, benefits and risks against participation and duration of the survey. Each participant has the right to agree or decline to participate in such survey.

### Variables included

Many variables were selected in this study. Age in five-year groups (15-19, 20-24, 25-29, 30-34, 35-39, 40-44 and 45-49), wealth index profile which was divided by the database collectors into five main categories (poorest, poorer, middle, richer and richest), highest educational level of the participants (no education, primary education, secondary education and higher education), urban or rural residence frequency of listening to radio (not at all, less than once a week and at least once a week), reading newspaper or magazine (not at all, less than once a week and at least once a week) and watching television (not at all, less than once a week and at least once a week).

### Statistical analysis

One of the authors analyzed the data by using SPSS version 24. A chi-square test was used according to the nature of the data which was categorical in nature and the data was reported in the results as percentage and frequency of each categorical variable at which the data was weighted before running the analysis. For identifying the predictors of HIV infection by witchcraft or supernatural means, we run a logistic regression model. The model was built as adjusted (multivariable) model for all potential covariates that were taken together in one model. We reported the logistic regression results by using the odds ratio (OR) and the corresponding 95% confidence interval (CI).

## Results

### Characteristics of participants

We included 15335 women, of those 620 (4%) thought that they can get HIV through witchcraft or supernatural means (Table 1). Most of the included sample was in the range of 15-29 years old that represented about two thirds of both the case and the control group. Around three quarters of the case group resided in rural areas compared to half of the case group. The majority of the case group (62.6%) had received no education while more than half of the control group had primary, secondary or higher education (55.6%). Regarding wealth index profile, 82.1% of the case group was categorized as poorest, poorer and middle; however, 63% of the control group wealth index was reported to be middle, richer and richest. The prevalence of the control group who listened to radio, read newspaper or magazine and watched television by at least once a week were higher than that of the case group (51.5% vs. 47.7%), (12.1% vs. 1.7%) and (67.2% vs. 40.6%), in order.

**Table 1:** Characteristics of the included women

	Variables	Controls = 14715 (96%)	Cases=620 (4%)	P value
**Age in 5-year groups (%)**	15-19	2956 (20.1)	198 (32)	**<0.001**
	20-24	2721 (18.5)	125 (20.2)	
	25-29	2562 (17.4)	90 (14.5)	
	30-34	2340 (15.9)	80 (12.3)	
	35-39	1688 (11.5)	63 (10.2)	
	40-44	1442 (9.8)	33 (5.3)	
	45-49	1005 (6.8)	31 (5)	
**Type of place of residence (%)**	Urban	7823 (53.2)	135 (21.9)	**<0.001**
	Rural	6892 (46.8)	485 (78.1)	
**Highest educational level (%)**	No	6380 (43.4)	389 (62.6)	**<0.001**
	Primary	3437 (23.4)	131 (21.1)	
	Secondary	4159 (28.3)	98 (15.9)	
	Higher	740 (5)	2 (0.4)	
**Wealth index combined (%)**	Poorest	2001 (13.6)	221 (35.7)	**<0.001**
	Poorer	2422 (16.5)	169 (27.3)	
	Middle	2956 (20.1)	118 (19.1)	
	Richer	3351 (22.8)	62 (11.2)	
	Richest	3984 (27.1)	42 (6.8)	
**Frequency of listening to radio (%)**	Not at all	2151 (14.6)	131 (21.1)	**0.001**
	Less than once a week	4953 (33.3)	194 (31.1)	
	At least once a week	7611 (51.1)	295 (47.7)	
**Frequency of reading newspaper or magazine (%)**	Not at all	10680 (72.6)	562 (90.6)	**<0.001**
	Less than once a week	2248 (15.3)	48 (7.7)	
	At least once a week	1787 (12.1)	10 (1.7)	
**Frequency of watching television (%)**	Not at all	2332 (15.9)	224 (36.1)	**<0.001**
	Less than once a week	2492 (16.9)	144 (23.3)	
	At least once a week	9891 (67.2)	252 (40.6)	
	Yes	7939 (58.9)	260 (35.3)	

### Multivariable analysis

In multivariable analysis, older age, receiving primary and secondary education, higher wealth index, more frequency of listening to radio, watching television for less than once a week and reading newspaper or magazine for at least once a week were significantly associated with a reduction in the witchcraft and supernatural means beliefs (p < 0.05) (Table 2). Moreover, rural residence was associated with an increase in the wrong HIV beliefs (p < 0.05).

**Table 2:** Predictors of beliefs about getting HIV infection by witchcraft or supernatural means

Variables		OR (95% CI)	P value
**Age in 5-year groups**	15-19	**Reference**	
	20-24	0.67 (0.54-0.83)	**<0.001**
	25-29	0.46 (0.36-0.59)	**<0.001**
	30-34	0.45 (0.35-0.58)	**<0.001**
	35-39	0.43 (0.33-0.57)	**<0.001**
	40-44	0.27 (0.19-0.39)	**<0.001**
	45-49	0.34 (0.23-0.48)	**<0.001**
**Highest educational level**	No	**Reference**	
	Primary	0.81 (0.66-0.99)	**0.039**
	Secondary	0.47 (0.37-0.60)	**<0.001**
	Higher	0.36 (0.11-1.20)	0.1
**Type of place of residence**	Urban	**Reference**	
	Rural	1.36 (1.10-1.68)	**0.004**
**Wealth index**	Poorest	**Reference**	
	Poorer	0.77 (0.64-0.93)	**0.01**
	Middle	0.55 (0.43-0.71)	**<0.001**
	Richer	0.39 (0.28-0.54)	**<0.001**
	Richest	0.27 (0.18-0.43)	**<0.001**
**Frequency of listening to radio**	Not at all	**Reference**	
	Less than once a week	0.77 (0.62-0.95)	**0.016**
	At least once a week	0.79 (0.65-0.97)	**0.02**
**Frequency of reading newspaper or magazine**	Not at all	**Reference**	
	Less than once a week	1.08 (0.79-1.50)	0.61
	At least once a week	0.44 (0.23-0.87)	**0.02**
**Frequency of watching television**	Not at all	**Reference**	
	Less than once a week	0.81 (0.66-0.99)	**0.038**
	At least once a week	0.80 (0.64-1.10)	0.055

## Discussion

Despite the efforts of scientists and caregivers in sensitizing the population about HIV transmission; misconceptions about this infection continue to exist in many countries 7-10. The poor knowledge and the existence of misconceptions about the transmission of HIV infection may lead to risky behaviors especially among HIV seropositive individuals 11. We aimed to provide evidence about the predictors of beliefs about getting HIV infection by witchcraft or supernatural means among Senegal women. These beliefs may represent a major barrier to the prevention and the management of this infection. Our findings showed a prevalence of wrong beliefs about getting HIV through witchcraft or supernatural means of 4% among Senegal women. Older age and receiving primary or secondary education were significantly associated with a reduction in witchcraft and supernatural means beliefs. Similar results were found in other African countries 12, 13. Educated people usually had more knowledge about HIV transmission, prevention or treatment. While non-educated people may not understand clearly the provided information in HIV prevention campaigns 14, 15. This observation should encourage policymakers to adapt their sensitization means in a manner that can be understood by all the categories of the population.

Listening to the radio or watching television and reading newspaper or magazine were associated with misconceptions reduction in our study. These findings are similar to other investigations done in China, Ghana, Nigeria, and Uganda 15-18. Mass media plays a crucial role in promoting health programs and in dispelling wrong ideas about human diseases. The large cross-sectional study from the DHS database across 13 Sub-Saharan countries demonstrated that media exposure enhance community knowledge about the infection and influence women's behavior regarding the prevention 19.

Furthermore, wealthy people have more access to education and preventive resources than poor people 20; this may explain the observed effect of higher wealth index in reducing witchcraft and supernatural means beliefs in our study. This finding is also supported by previous studies conducted in Bangladesh, Ghana, Malawi and Ethiopia 7, 9, 20-22. The inadequate knowledge observed in middle-and low-income countries can lead to decreased usage of HIV-prevention ways resulting in risky behaviors 22.

Moreover, since the level of education, media exposure and wealth index can influence people's perspectives about HIV dissemination and prevention, the lack of resources and of education can have a considerable impact on the spread of misconceptions in rural regions in sub-Saharan African countries. The later is associated with poverty and low educational level 20. To our knowledge, rural residents had less access to media and less knowledge about many diseases compared to urban ones 23. This evidence has been shown in other studies in some countries including Canada 24 and South Africa 25.

Our study encourages policymakers in Senegal to take steps forward in understanding the factors that promote the spread of wrong ideas about HIV and to apply the appropriate measures such as increasing the awareness of the infection transmission, symptoms and treatment, increasing the educational level especially in the rural regions as well as increasing –whenever available- the socio-economic status in poor families, in facing these misconceptions and in promoting HIV prevention in the region. However, as noted, a big relationship may exist between urbanization, education, the availability of media, and the other socioeconomic factors.

We faced many limitations that needed to be overcome by future research. This study is a survey in which the data is self-reported. This type of scientific investigations may be sometimes subject to bias, especially when it interests sensitive topics like HIV infection. Yet, our findings are consistent with previous findings and can contribute in guiding policymakers in their decision regarding the prevention of HIV spread. Moreover, we do not have the information about HIV status of the participants or their relatives which could play a substantial role regarding the HIV misconceptions.

## Conclusion

We demonstrated many predictors of the wrong beliefs about getting HIV infection by witchcraft or supernatural means in the Senegalese women. Policymakers should initiate health educational programs in parallel with increasing the socioeconomic status especially in women who are at high risk of having these wrong beliefs to limit the HIV transmission. In addition, continuous monitoring of the HIV knowledge in the endemic countries is crucial to decrease HIV burden.

## Data Availability

The data that supports the findings of this study are available from the corresponding author upon reasonable request.

## References

[1] https://www.who.int/teams/global-hiv-hepatitis-and-stis-programmes/hiv/strategic-information/hiv-data-and-statistics.

[2] Frank TD, Carter A, Jahagirdar D, Biehl MH, Douwes-Schultz D, Larson SL (2019). Global, regional, and national incidence, prevalence, and mortality of HIV, 1980–2017, and forecasts to 2030, for 195 countries and territories: a systematic analysis for the Global Burden of Diseases, Injuries, and Risk Factors Study 2017. The Lancet HIV.

[3] Haas AD, Zaniewski E, Anderegg N, Ford N, Fox MP, Vinikoor M (2018). Retention and mortality on antiretro-viral therapy in sub-Saharan Africa: collaborative analyses of HIV treatment programmes. Journal of the International AIDS Society.

[4] Tenkorang EY, Gyimah SO, Maticka-Tyndale E, Adjei J (2011). Superstition, witchcraft and HIV prevention in sub-Saharan Africa: the case of Ghana. Culture, health & sexuality.

[5] Kalichman SC, Simbayi L (2004). Traditional beliefs about the cause of AIDS and AIDS-related stigma in South Africa. AIDS care.

[6] Hamra M, Ross MW, Orrs M, D'Agostino A (2006). Relationship between expressed HIV/AIDS-related stigma and HIV-beliefs/knowledge and behaviour in families of HIV infected children in Kenya. Tropical Medicine & International Health.

[7] Sano Y, Antabe R, Atuoye KN, Hussey LK, Bayne J, Galaa SZ (2016). Persistent misconceptions about HIV transmission among males and females in Malawi. BMC international health and human rights.

[8] Kambutse I, Igiraneza G, Ogbuagu O (2018). Perceptions of HIV transmission and pre-exposure prophylaxis among health care workers and community members in Rwanda. PloS one.

[9] Seid A, Ahmed M (2020). What are the Determinants of Misconception About HIV Transmission Among Ever-Married Women in Ethiopia?. HIV/AIDS (Auckland, NZ).

[10] Antabe R, Sano Y, Anfaara FW, Luginaah I (2021). Reducing HIV misconceptions among females and males in Malawi: are we making progress?. AIDS care.

[11] Gerbi GB, Habtemariam T, Tameru B, Nganwa D, Robnett V (2012). A quantitative risk assessment of multiple factors influencing HIV/AIDS transmission through unprotected sex among HIV-seropositive men. AIDS care.

[12] Asaduzzaman M, Higuchi M, Sarker MAB, Hamajima N (2016). Awareness and knowledge of HIV/AIDS among married women in rural Bangladesh and exposure to media: a secondary data analysis of the 2011 Bangladesh Demographic and Health Survey. Nagoya journal of medical science.

[13] Ciampa PJ, Skinner SL, Patricio SR, Rothman RL, Vermund SH, Audet CM (2012). Comprehensive knowledge of HIV among women in rural Mozambique: development and validation of the HIV knowledge 27 scale. PloS one.

[14] Chege F, Sifuna DN (2006). Girls' and women's education in Kenya. Gender Perspectives and trends.

[15] Li L, Rotheram-Borus MJ, Lu Y, Wu Z, Lin C, Guan J (2009). Mass media and HIV/AIDS in China. Journal of health communication.

[16] Benefo KD (2004). The mass media and HIV/AIDS prevention in Ghana. Journal of Health and Population in Developing Countries.

[17] Keating J, Meekers D, Adewuyi A (2006). Assessing effects of a media campaign on HIV/AIDS awareness and prevention in Nigeria: results from the VISION Project. BMC Public health.

[18] Bessinger R, Katende C, Gupta N (2004). Multi-media campaign exposure effects on knowledge and use of condoms for STI and HIV/AIDS prevention in Uganda. Evaluation and program planning.

[19] Jung M, Arya M, Viswanath K (2013). Effect of media use on HIV/AIDS-related knowledge and condom use in sub-Saharan Africa: a cross-sectional study. PloS one.

[20] Tenkorang EY (2013). Myths and misconceptions about HIV transmission in Ghana: what are the drivers?. Culture, health & sexuality.

[21] Mondal MNI, Hoque N, Chowdhury MRK, Hossain MS (2014). Factors associated with misconceptions about HIV transmission of ever-married women in Bangladesh. Japanese journal of infectious diseases.

[22] Yang F, Li Z, Subramianian S, Lu C (2021). Assessment of Knowledge of HIV/AIDS and Association with Socio-economic Disparities Among Young Women in Low-and Middle-Income Countries, 2003 to 2018. JAMA network open.

[23] Warner ES, Murray AD, Palmour VE (1973). Information needs of urban residents.

[24] Veinot TC, Harris R (2011). Talking about, knowing about HIV/AIDS in Canada: A rural-urban comparison. The Journal of Rural Health.

[25] Peltzer K, Matseke G, Mzolo T, Majaja M (2009). Determinants of knowledge of HIV status in South Africa: results from a population-based HIV survey. BMC public health.

